# Uncovering Benzene Pollution Patterns Using an Interpretable, Setting-Aware Artificial Intelligence Approach

**DOI:** 10.3390/toxics14020181

**Published:** 2026-02-18

**Authors:** Ivan Bešlić, Timea Bezdan, Gordana Jovanović, Silvije Davila, Gordana Pehnec, Snježana Herceg Romanić, Andreja Stojić, Mirjana Perišić

**Affiliations:** 1 Institute for Medical Research and Occupational Health, Ksaverska cesta 2, P.O. Box 291, 10001 Zagreb, Croatia; ibeslic@imi.hr (I.B.); sdavila@imi.hr (S.D.); gpehnec@imi.hr (G.P.); sherceg@imi.hr (S.H.R.); 2 Faculty of Informatics and Computing, Singidunum University, Danijelova 32, 11000 Belgrade, Serbia; tbezdan@singidunum.ac.rs; 3 Institute of Physics Belgrade, National Institute of the Republic of Serbia, Pregrevica 118, 11080 Belgrade, Serbia; andreja.stojic@ipb.ac.rs (A.S.); mirjana.perisic@ipb.ac.rs (M.P.); 4 Environment and Sustainable Development Studies, Singidunum University, Danijelova 32, 11000 Belgrade, Serbia

**Keywords:** air pollution, gaseous pollutants, machine learning, metaheuristics, explainable artificial intelligence

## Abstract

We investigated benzene variability in an urban environment using an interpretable, setting-based artificial intelligence framework. A seven-year dataset (2017–2023) of hourly pollutant concentrations (benzene, NO_2_, SO_2_, CO, O_3_) measured in Zagreb (Croatia) was analyzed, as were meteorological variables. Multiple-ensemble decision tree models were developed, with hyperparameters optimized using metaheuristic algorithms. The best-performing model, Extra Trees optimized by the Sine Cosine Algorithm, achieved an R^2^ of 0.87. Model interpretation employed Shapley additive explanations (SHAP), followed by PaCMAP embedding and HDBSCAN clustering to identify coherent environmental settings. Seven settings (C0–C6) and one residual group were identified, representing pollution-enhancing, suppressing, and transitional regimes. Two settings dominated benzene extremes. C6 reflected winter stagnation, characterized by strong combustion influence (CO contribution of 11.9%), shallow boundary layers (~290 m), weak winds, and high humidity. C4 represented a synoptic stability regime with enhanced heat fluxes and diminished after the COVID-19 period, consistent with altered anthropogenic activity. Low-benzene settings (C0, C1, C3) were associated with stronger mixing and higher oxidizing capacity, while transitional settings (C2, C5) reflected moderate conditions. Overall, the results show that a small number of environmental settings governed the benzene extremes, providing a transferable and interpretable framework for air quality assessment and policy support.

## 1. Introduction

Urban air pollution, driven by intensified industrial activity, dense transportation networks, and rapid urbanization, is one of the leading environmental risk factors worldwide, responsible for several million premature deaths annually [1]. Among atmospheric pollutants, particulate matter, ground-level ozone, sulfur and nitrogen oxides, and selected volatile organic compounds (VOCs) are recognized as key contributors to morbidity and premature mortality [2]. Although causal evidence is strongest for particulate matter and ozone [3], several VOCs, including benzene, are toxic or carcinogenic and therefore require continued regulatory oversight and scientific investigation.

Benzene’s behavior in the atmosphere is governed not only by direct anthropogenic emissions, primarily traffic, industrial processes, fuel evaporation, solvent use, and biomass burning, but also by its interaction with co-pollutants and meteorological processes governing dispersion, mixing, and photochemical degradation [4,5]. These interactions can vary drastically across environmental settings (ESs), such as wintertime stagnation and inversion conditions, or summertime photochemically active periods [6]. Under such settings, the same driver, e.g., wind speed, boundary-layer height, or co-pollutant concentration, can exert qualitatively different, often nonlinear influences on benzene. Such complex influences suggest that benzene should be understood not as an isolated pollutant, but within an interrelated environmental context where emissions, chemistry, meteorology, orography, other environmental factors, and human activity jointly define its variability.

Traditional tools for studying air pollutant dynamics, chemical transport models (CTMs) and statistical frameworks have offered valuable mechanistic and empirical insights. However, CTMs are sensitive to uncertainties in emission inventories, numerical parameterizations, chemical mechanisms, meteorological fields, especially boundary-layer processes, and others [7]. Conversely, conventional statistical models often fail to capture nonlinear and setting-dependent pollutant responses to multiple environmental and socio-economic drivers. These limitations motivate the need for approaches capable of capturing emergent, interaction-driven structures in atmospheric systems, particularly under rapidly evolving urban emission profiles and growing environmental complexity.

Recent advances in artificial intelligence (AI) have accelerated a paradigm shift in atmospheric sciences. Machine learning (ML) models, built on integrated heterogeneous data, now routinely learn complex, nonlinear relationships between meteorology, emissions and air quality, and support real-time forecasting and mapping [8]. The next-generation environmental analytics increasingly emphasize interpretation of ML models, where hybrid physics-data paradigms and explainable frameworks uncover actionable environmental insights [9,10]. In this context, the model interpretation bridges a critical methodological gap by enabling pollutant dynamics to be analyzed as emergent outcomes of environmental regimes rather than as direct responses to individual emission sources. These regimes are represented as ES, coherent multivariate states shaped by combined influence of atmospheric chemistry, meteorology, temporal patterns and the broader emission environment.

Building on this perspective, the concept of ES has recently been introduced as an outcome of an AI-driven framework for characterizing the atmospheric fate of pollutants [4]. Briefly, an ES denotes the ensemble of co-occurring natural and anthropogenic conditions, including meteorology, temporal structure, co-pollutant levels, orographic features or human activity, that jointly govern pollutant behavior. Embedding pollutant concentrations within this broader constellation of meteorological and socio-environmental drivers moves beyond conventional source apportionment and provides a more robust basis for characterizing emissions, sinks, and context-dependent effects. Crucially, it makes explicit that the influence of any given variable (e.g., temperature or wind speed) is contingent on the surrounding state of the system.

In this paper, we used an AI-based framework to investigate the ES that govern benzene dynamics in the urban atmosphere using primarily routinely regulated monitoring data. We trained metaheuristically optimized decision tree ensembles to predict benzene concentrations from commonly measured criteria gaseous co-pollutants (O_3_, CO, SO_2_, NO_2_) and meteorological parameters derived from the Global data assimilation system (GDAS1) reanalysis over a seven-year observation period. The best performing model was then interpreted using SHapley Additive exPlanations (SHAP) [11] and the resulting impacts were grouped using Hierarchical density-based spatial clustering of applications with noise (HDBSCAN) [12] to reveal dominant ES. Recent ML studies have highlighted that robust generalization under limited and imperfect monitoring data requires approaches that explicitly extract structure rather than relying on dense datasets [13], reinforcing the relevance of the interpretable, setting-based framework adopted here.

Specifically, we aimed to (1) identify the key ES impacting benzene variability, (2) quantify the relative influence of routinely monitored co-pollutants and meteorological drivers on benzene behavior, and (3) demonstrate the capability of AI to uncover nonlinear pollution settings overlooked by conventional methods. By relying on a limited but widely measured predictor set, this work demonstrates how robust, setting-based insights can be extracted from standard regulatory measurements, offering a scalable and transferable framework for understanding pollutant dynamics in data-limited environments, even when long-term observations originate from a single monitoring site. Building on our earlier introduction of the ES concept [4], the present work advances the framework from an event-driven, data-rich demonstration toward a long-term, monitoring-compatible methodology. Whereas the previous study was based on a short-term (2 March–15 May 2020), high-resolution VOC dataset acquired using non-standardized instrumentation and explicit human-activity indicators during the highly perturbed COVID-19 period in Belgrade (Serbia), the current analysis relies exclusively on routinely monitored criteria pollutants and meteorological variables collected over a seven-year period (2017–2023) in Zagreb, Croatia. This shift explicitly tests the robustness of the ES approach under realistic regulatory monitoring constraints and aims to identify physically interpretable regimes that can be resolved without specialized instrumentation or exogenous socio-economic datasets. The extended temporal coverage enables the identification of persistent, recurrent, and transitional settings spanning seasons, emission phases, and pre- and post-COVID conditions, thereby moving beyond case-specific responses toward regime-level characterization. The emergence of coherent, process-consistent settings in a different city, climatic setting, and emission context supports the transferability of the framework across contrasting data environments, while acknowledging that systematic multi-site validation remains a necessary next step for operational and policy-oriented applications.

## 2. Materials and Methods

### 2.1. Data Collection

This study used air quality and meteorological data collected from 2017 to 2023 at the Ksaverska cesta monitoring station, situated in the northern residential sector of Zagreb, the capital of Croatia (45°50′09″ N, 15°58′59″ E). The station is classified as an urban background site and is representative of a moderately populated residential area. The monitoring location is positioned approximately 30 m from a roadway characterized by moderate traffic intensity, ensuring that the measured concentrations reflect combined influences of residential heating emissions and background urban traffic.

Hourly concentrations of benzene, NO_2_, CO, SO_2_, and O_3_ were quantified at the monitoring site using standardized reference methods EN 14662-3 [14], EN 14211 [15], EN 14626 [16], EN 14212 [17], and EN 14625 [18]. All measurements were performed by an accredited laboratory operating in accordance with EN ISO/IEC 17025 [19], as part of the local air quality network supported by the City of Zagreb. Prior to modeling, pollutant records underwent a preprocessing in which atypical values (likely arising from instrumental malfunctions or reading inconsistencies) were removed, and all incomplete observations were excluded. This procedure yielded a consistent and reliable dataset, consisting of 39,124 complete hourly records.

To provide comprehensive meteorological context, air quality observations were supplemented with more than 20 surface-level meteorological variables obtained from the GDAS1 operated by NOAA (Table S1). GDAS1 provides three-hourly global fields generated through combined observational datasets and numerical weather prediction. Meteorological data were linearly interpolated to an hourly time step to enable seamless integration with the pollutant dataset. The final modeling dataset comprised five gaseous pollutants (including benzene as the target variable) and 29 meteorological predictors, yielding a comprehensive set of environmental features suitable for detailed, fine-scale analysis of benzene variability under diverse atmospheric conditions.

### 2.2. Data Analysis

The ensemble-based ML framework adopted in this study follows the principles described by Dietterich [20] and was implemented primarily in Python (v3.x) [21]. Model development and evaluation were performed using the scikit-learn library (v1.4), together with CatBoost (v1.2.7), LightGBM (v4.5.0), and XGBoost (v2.1.3). Hyperparameter optimization was conducted using metaheuristic algorithms implemented in the Mealpy framework (v3.0.1). Model interpretability and post hoc analysis were carried out using SHAP (v0.46.0) and Shapley Additive Global importance, SAGE (v0.2.4). Dimensionality reduction was performed using PaCMAP [22], while clustering was carried out using HDBSCAN (v0.8.40). Basic statistical analyses and data consistency checks were additionally performed using R (version 4.x) [23].

Benzene concentrations were selected as the target variable, while gaseous pollutants and meteorological parameters served as predictors within the AI-framework that explicitly aims to capture complex, interaction-driven structures and to represent benzene dynamics as emergent outcomes of multivariate ES. The analytical workflow combined ensemble decision tree regression modeling, model hyperparameter optimization using metaheuristic, explainable AI (XAI) to explain the best performing model, and postprocessing of the XAI outputs to achieve both predictive skill and physical interpretability [4]. Because the primary objective was not operational forecasting but examination of the statistical and structural properties of the derived ES, the main evaluation relied on randomized partitioning rather than strict chronological forecasting splits. However, to assess robustness against potential temporal leakage and autocorrelation effects, an additional forward-chaining time-series validation (5 folds using TimeSeriesSplit) was conducted as a sensitivity analysis. Under randomized 80/20 partitioning, the Extra Trees model achieved an R^2^ of 0.87 on the independent test set. Under forward-chaining validation, the resulting R^2^ scores across folds were 0.66, 0.68, 0.70, 0.63, and 0.29, with a mean R^2^ of 0.59. Although lower than the randomly partitioned evaluation, these results demonstrate that substantial explanatory power persists under strict chronological separation, indicating that the reported performance and the derived ES structures are not solely artifacts of temporally intermingled sampling.

Missing values were excluded due to their limited occurrence and the exploratory scope of the analysis. Feature scaling was not performed, as ensemble tree-based models are invariant to monotonic transformations and do not require normalization. Briefly, the tested ensemble models included ExtraTrees [24], Gradient Boosting and its histogram-based variant [25], LightGBM [26], and XGBoost [27]. After baseline evaluation, the three best-performing models were further optimized using two metaheuristic strategies, the Harris Hawks Optimization [28] and Sine Cosine Algorithm [29].

For each of the three best-performing models (Extra Trees, LightGBM, and XGBoost), bounded search spaces were defined over key structural hyperparameters and optimized as a maximization problem using mean cross-validated R^2^ (5-fold KFold with shuffle enabled and random_state = 42, such that in each fold approximately 80% of the training data were used for model fitting and 20% for validation). Each metaheuristic run was conducted for 10 epochs with a population size of 20 and a fixed random seed (42), ensuring deterministic reproducibility of the optimization procedure.

For the Extra Trees model, the number of estimators was searched within the range 10–1000, maximum tree depth within 1–100, the fraction of features considered at each split within 0.1–1.0, minimum samples required for splitting within 2–10, and minimum samples per leaf within 1–10. For LightGBM, the number of leaves was optimized within 10–300, maximum depth within 10–100, learning rate within 0.001–0.2, number of boosting estimators within 100–2000, subsample fraction within 0.6–1.0, column sampling fraction within 0.6–1.0, and L1 and L2 regularization parameters (reg_alpha and reg_lambda) within 0–1. For XGBoost, the search space included maximum depth (3–10), learning rate (0.001–0.3), number of estimators (100–1000), subsample ratio (0.1–1.0), column sampling ratio (0.1–1.0), L1 and L2 regularization terms (0–10), gamma (0–10), and minimum child weight (0–10).

Metaheuristic optimization was applied to explore model sensitivity and achievable performance gains under computationally feasible constraints rather than to define an exhaustively optimized final model. Model performance was assessed using the coefficient of determination (R^2^). After selecting the best hyperparameter configuration, the final model was refitted on the full 80% training subset and evaluated on the independent 20% test set obtained via a random 80/20 partition using train_test_split (shuffle = True, random_state = 42).

The top-ranked model was interpreted using SHAP, which provides predictor-specific contributions to individual predictions and thus enables a localized characterization of predictor–response relationships across the full range of observed conditions. SHAP-based interpretability analyses were conducted exclusively on the held-out test subset to ensure that explanation patterns were derived from unseen data. To improve interpretability, we derived additional SHAP-based indicators, including relative SHAP values that express each variable’s contribution as a fraction of the total model attribution [30], as well as normalized SHAP values referenced to the model’s expected prediction. In this paper, we refer to mean impact as the mean value of SHAP values, mean absolute impact as the mean value of absolute SHAP values, mean relative impact as the mean value of relative SHAP values, and mean absolute normalized impact as the mean value of absolute normalized SHAP values.

To further structure these high-dimensional explanation patterns in a way consistent with the ES concept, SHAP values were embedded using Pairwise controlled manifold approximation (PaCMAP) [22] and clustered with HDBSCAN. The resulting clusters in SHAP space were interpreted as ES, i.e., coherent multivariate atmospheric states defined jointly by co-occurring environmental conditions. At the cluster level, setting frequency, mean SHAP value, and relative contribution to benzene predictions were analyzed to quantify prevalence and strength of influence. At the variable level, characteristic predictor values within each setting were evaluated alongside their SHAP impacts, linking dominant co-pollutant and meteorological conditions to modeled benzene variability. This integrated evaluation provides a consistent basis for interpreting ES and elucidating mechanisms governing benzene fluctuations in the urban atmosphere.

## 3. Results and Discussion

### 3.1. Descriptive Statistics

Table S2 summarizes the descriptive statistics of major air pollutants and meteorological variables. All primary pollutants remained below regulatory limits (Directive 2008/50/EC; Directive 2024/2881/EC), whereas O_3_ frequently exceeded the 8 h target value, underscoring persistent challenges related to secondary pollution. Over 2017–2023, the mean benzene concentration was 1.02 µg m^−3^, with episodic peaks up to 13.52 µg m^−3^, indicating intermittent emission events. The highest benzene concentrations (12.25–13.52 µg m^−3^) occurred predominantly between 2017 and 2020, whereas after 2020 peak values generally declined and remained below 10 µg m^−3^ (Table S3). Several of these extreme values were associated with wintertime ES characterized by inversion and stagnation with strong combustion influence, as discussed below. In contrast, part of the extreme and atypical cases remained unclustered and were assigned to the residual C-1 group, reflecting heterogeneous conditions with limited physical interpretability. NO_2_ averaged 16.8 µg m^−3^, decreasing steadily from 2017 to 2023, while CO showed a similar declining trend (0.36 to 0.28 mg m^−3^), consistent with reduced combustion-related emissions. SO_2_ exhibited generally low levels but with occasional sharp peaks, including a temporary increase in 2023 likely linked to nearby construction activities. O_3_ showed the greatest variability, with a mean of 54.1 µg m^−3^ and maxima up to 249.9 µg m^−3^, reflecting strong photochemical activity during warm, stable conditions.

Interannual trends (Table S3) revealed declining benzene, NO_2_ and CO concentrations, consistent with reduced anthropogenic activity, particularly during and after the COVID-19 period. Studies carried out in Zagreb showed a pronounced decrease in NO_2_ levels during the COVID-19 lockdown. However, the significant decline was not observed for some other pollutants such as particulate matter and PAHs [31,32]. In contrast, O_3_ peaked in 2021–2022, reflecting meteorological conditions favorable for photochemical formation, in line with previous regional studies. These peaks likely occurred during periods characterized by elevated temperatures, intense solar radiation, and stable atmospheric stratification, meteorological regimes that are well-known to enhance photochemical O_3_ production and have recently been reported across Europe during summer heatwaves [33]. The pronounced seasonal and interannual variability of ozone concentrations is consistent with previous findings at this site for the period 2003–2016 [34]. Benzene decreased from 1.50 µg m^−3^ in 2018 to 0.63 µg m^−3^ in 2022, before rising again in 2023, indicating episodic reintensification of emissions.

In addition to interannual variability, benzene exhibited pronounced temporal structure across multiple timescales (Figure S1). Diurnal profiles revealed elevated concentrations during the morning and evening hours, consistent with increased urban activity and traffic intensity, while lower levels were observed during nighttime and early afternoon. Weekly patterns showed only modest weekday–weekend differences, reflecting the urban background character of the monitoring site. In contrast, seasonal variability was strong, with the highest benzene levels occurring in winter and the lowest in summer, highlighting the dominant role of atmospheric stability, boundary-layer dynamics, and photochemical removal.

Meteorological conditions featured low wind speeds, frequent stability, and variable boundary layer heights (Table S2), favoring pollutant accumulation and photochemical processing. Together, these patterns highlighted the need for analytical approaches that go beyond compliance metrics and capture the coupled roles of emissions, meteorology, and chemistry, providing the rationale for the setting-based AI framework applied in this study.

### 3.2. Environmental Settings

Among the best performing ML models, the Extra Trees optimized with the SCA achieved the best performance, with a coefficient of determination R^2^ = 0.87. The SHAP value clustering produced seven ES (C0–C6) and one unclustered group (C-1) (Table 1). The C-1 group comprised 33.5% of the data. It was characterized by a mean benzene concentration close to the overall average, with a negligible mean impact of predictors. However, the large mean absolute normalized impact (132.63%) indicates strong but opposing SHAP contributions that cancel when averaged, consistent with heterogeneous or transitional conditions in which competing emission, dispersion, and chemical processes act simultaneously rather than forming a stable regime. The C-1 group also includes unclustered rare or mixed states that occur too infrequently or lack sufficient similarity to define a coherent setting. Such behavior is common in long-term urban datasets and reflects the limits of regime-based abstraction for highly transient conditions. Future methodological refinements, including ML approaches designed to improve robustness under sparse or extreme conditions [13], may help reduce the residual group and further resolve transitional regimes. Although the residual group (C-1) accounts for approximately one-third of the observations, it is dominated by near-average benzene concentrations (Table 1) and therefore does not contribute to the high-concentration regimes that are relevant for exposure assessment or regulatory considerations. Accordingly, all policy-relevant conclusions in this study are driven by the well-defined environmental settings (C0–C6), whereas C-1 primarily reflects transitional or heterogeneous states that fall outside stable regime abstraction in long-term urban datasets.

The distribution of ES was highly uneven, both in frequency and in their influence on benzene variability (Table 1 and Table S4). Two settings, C6 and C0, were most frequent (each approximately 17%) but represented contrasting conditions. C6 was strongly pollution-enhancing, characterized by the highest mean benzene concentration (2.02 µg m^−3^), extreme values (up to 12.96 µg m^−3^), and a dominant positive contribution (97.7%). In contrast, C0 reflected a dilution-dominated setting with much lower concentrations (mean: 0.44 µg m^−3^, maximum: 4.96 µg m^−3^) and a strong negative influence, effectively suppressing benzene levels. Less frequent settings (C1, C2, C3, and C5) together accounted for under 20% of occurrences and were generally associated with low-to-moderate benzene levels. C1 exhibited the strongest suppression, while C2 and C3 occasionally produced short-lived peaks (up to 5.44 in C3) despite overall low means, indicating episodic accumulation under otherwise dispersive conditions. C5 showed relatively stable, moderate concentrations. Setting C4, although less frequent, represented another pollution-enhancing setting, with elevated concentrations up to 5.13 µg m^−3^ and positive impacts, albeit weaker than C6.

**Figure 1:**
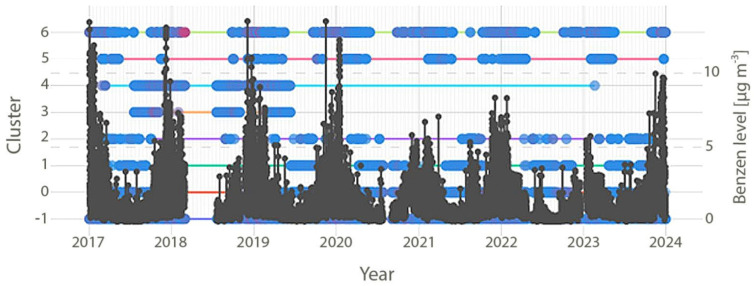
Temporal distribution of environmental settings (C1–C6) and benzene levels [µg m^−3^].

Temporally, settings were clustered rather than randomly distributed (Figure 1). High-impact settings (C6, C4) dominated earlier years, whereas lower-impact settings became more prevalent after 2020. This pattern suggests that benzene extremes arise from a limited number of recurring environmental configurations and that shifts in their frequency, rather than changes in average concentrations, drive long-term variability.

#### 3.2.1. Pollution-Enhancing Environmental Settings: Winter Inversions and Pre-COVID Heat Flux Settings

C6 and C4 emerged as the dominant pollution-enhancing settings, each shaped by distinct combinations of emission processes and meteorological conditions. Setting C6 was associated with stagnant wintertime episodes and combustion-related sources, whereas C4 reflected pre-COVID conditions characterized by structurally different anthropogenic emission patterns, enhanced heat fluxes, and shallower boundary layers.

##### Setting C6—Winter Inversion–Stagnation with Strong Combustion Influence and Extreme Benzene Episodes

Within C6 (Table S5 and Figure 1), the SHAP values identified CO as the dominant predictor of benzene variability, with a mean relative impact of 11.9%. Although its mean concentration remained modest (0.5 mg m^−3^), values occasionally exceeded 2.8 mg m^−3^, closely tracking benzene due to their shared combustion origin [35]. In contrast, O_3_ (normalized impact 0.5%), NO_2_ (−1.2%), and SO_2_ (−2.6%) contributed less or negatively. The occasional maxima of O_3_ (137 µg m^−3^) and SO_2_ (19.5 µg m^−3^) likely reflected episodic regional photochemical production or short-lived contributions from nearby point sources, respectively. However, these inputs do not translate into sustained benzene enhancement under winter stagnation.

Meteorological factors further reinforced benzene accumulation in C6 with concentrations peaks up to 12.96 µg m^−3^ (Table S5). Positive normalized SHAP contributions arose from near-surface temperature (7.1%), latent heat flux (2.2%), and boundary-layer indicators such as surface temperature (1.0%) and the standard lifted index (0.8%) (Table S5). These were consistent with stagnant winter episodes characterized by shallow boundary layers (mean height of 290 m), low wind speeds (1.58 m s^−1^), high relative humidity (84.6%), and low near-surface temperatures (mean 3.5 °C, with episodic minima down to −14 °C and maxima up to +16 °C). Such stagnant conditions suppressed vertical mixing and advection, locking pollutants near the surface. Winter inversions, in which temperature increases with altitude, further limited benzene dispersion [36,37].

From a chemical perspective, prolonged benzene lifetime may reflect suppression of its dominant gas-phase sink, oxidation by hydroxyl radicals (•OH), which controls benzene removal on urban timescales [38]. Elevated relative humidity can indirectly reduce •OH availability by modifying radical budgets through enhanced heterogeneous and aqueous-phase processes, including uptake of •OH precursors and radical scavenging on moist aerosol or liquid water surfaces [39]. In winter, high humidity is typically coupled with reduced solar radiation and weakened photochemistry, further constraining •OH production and overall oxidative capacity. Although direct experimental evidence specifically linking humidity to benzene lifetime remains limited, the broader atmospheric chemistry literature consistently indicates constrained oxidant availability and slower VOC oxidation under humid, low-radiation winter environments [40,41]. Because oxidant concentrations were not measured directly in this study, changes in oxidative capacity cannot be quantified explicitly. Instead, routinely monitored co-pollutants and meteorological variables act as indirect indicators of combustion intensity, mixing, and photochemical conditions. Accordingly, the inferred reduction in benzene oxidation under humid, stagnant conditions should be interpreted as a mechanistic hypothesis consistent with established atmospheric chemistry rather than a directly resolved causal pathway.

Although C6 captures these winter stagnation states, it is not homogeneous. Two subsettings were identified (Table S12). Subsetting C6-S0 was marked by the highest benzene concentrations (3.23 µg m^−3^ and mean relative impact of 2.2 µg m^−3^), making it the dominant high-exposure state (core winter pollution peak). In contrast, subsetting C6-S1 (14.7% of instances) corresponded to cleaner episodes (0.57 µg m^−3^ and negative impact −0.43 µg m^−3^), showing that C6 encompassed both severe pollution peaks and occasional diluted phases that occurred within the same broad meteorological envelope (diluted winter stagnation). Overall, C6 was governed by the heavily polluted subsetting C6-S0, emphasizing the interplay of combustion emissions and stagnant winter meteorology as the primary driver of extreme benzene episodes.

##### Setting C4—Pre-COVID Accumulation with Enhanced Heat Fluxes and Shallow Mixing

Unlike C6, which is tightly tied to winter stagnation and combustion emissions, C4 occurs throughout the year. The setting is shaped primarily by meteorological drivers that favor pollutant accumulation under slightly warmer and drier conditions. In C4, among all variables, latent heat flux emerged as the most influential positive predictor, with a mean relative impact of 1.7% (Table S6). Meteorological conditions within this setting included weak winds (1.6 m s^−1^), high humidity (about 83%, occasionally approaching saturation), and moderate near-surface temperatures (7.9 °C, with excursions up to 24 °C). Such stagnant settings suppressed dispersion and dilution, enabling benzene accumulation like in C6 but under somewhat warmer and seasonally broader contexts.

Negative SHAP contributions further highlighted the moderating role of atmospheric processes. The standard lifted index (−1.0%), soil moisture (−1.4%), and downward shortwave radiation (−1.7%) exerted consistent negative impacts. Downward shortwave radiation showed maximum positive excursions above 11%, suggesting that under sunny conditions enhanced photochemistry can accelerate benzene degradation, counteracting accumulation. Such sensitivity of oxidant production to solar radiation and boundary-layer dynamics is consistent with recent analyses of O_3_ and oxidant budgets in polluted environments [42].

Co-pollutants reinforced this interpretation. CO, with a mean level of 0.34 mg m^−3^ and a mean relative impact of 1.6%, reflected its role as a combustion co-emission with benzene. In contrast, NO_2_ (−0.4%) and SO_2_ (−0.2%) contributed negatively on average, with occasional peaks of their concentrations up to 75 µg m^−3^ and 29 µg m^−3^, respectively. These episodes suggested that although these pollutants can co-occur within combustion activity, their variability in C4 was not tightly coupled to benzene.

Subclustering revealed the internal heterogeneity of C4. Subclusters C4-S0 and C4-S-1 both produced strong positive impacts, elevating benzene to 1.65–1.81 µg m^−3^ (65–76% above the expected value; pre-COVID accumulations). By contrast, C4-S1 presented much lower concentrations (0.45 µg m^−3^) and negative contributions, capturing cleaner but less frequent states (clean pre-COVID). Since C4 was present year-round, this internal heterogeneity may have reflected how similar meteorological regimes and emission signals manifested differently across seasons. Overall, C4 functioned as a pollution-enhancing state, but its role was shaped by the balance between subclusters that either amplified or mitigated accumulation.

The disappearance of C4 after 2020 coincided with the onset of the COVID-19 pandemic, when traffic, industrial activity, and other urban emissions sharply declined in Zagreb [31]. C4 occurred across all seasons primarily sustained by meteorological conditions that favored accumulation under moderate temperatures and weak dispersion. Its absence after 2020 therefore cannot be attributed to seasonal variability. Rather, it reflects the structural shift in anthropogenic emissions, particularly from traffic-related sources, that previously supplied sufficient benzene and co-emitted precursors for accumulation under stagnant conditions (Figure S1). Although the meteorological drivers, characteristics of C4 (weak winds, high humidity, and suppressed mixing) continued to occur, the reduced availability of combustion-related inputs was no longer sufficient to sustain the elevated benzene levels associated with this setting.

#### 3.2.2. Pollution-Suppressing Environmental Settings: Photochemical Loss and Vertical Mixing

Settings C1, C0, and C3 corresponded to atmospheric conditions dominated by dispersion, dilution, and enhanced oxidative capacity, in which anthropogenic inputs were weaker, and meteorological drivers played a central role in lowering near-surface benzene levels.

##### Setting C1—Clean, Well-Ventilated Spring–Summer Background with Efficient Photochemical Loss

Setting C1, which accounted for 8.8% of all instances, emerged as the strongest suppressor, consistently associated with the lowest benzene levels (mean 0.30 µg m^−3^ and maximum 1.44 µg m^−3^). It represented a clean, well-ventilated atmospheric state that systematically drove concentrations downward and predominantly occurs from late spring through early autumn (April/May–October/November; Figure 1).

In this setting, CO (−37%) and near-surface air temperature (−12.7%) were the predictors with the strongest negative normalized impacts, followed by NO_2_ (−6.4%), O_3_ (−4.2%), SO_2_ (−4.1%), and the lifted index (−3.4%) (Table S7). These patterns indicated that suppression in C1 arose from weak combustion-related inputs and efficient atmospheric removal. The strong negative contribution of CO was consistent with low mean concentrations (0.17 mg m^−3^), reflecting reduced traffic and heating activity, while elevated temperatures (mean 15.8 °C, occasionally exceeding 24 °C) promoted deeper boundary-layer development and enhanced photochemical activity, increasing oxidant production and accelerating benzene degradation, in line with the established role of •OH-driven oxidation under warm, sunlit conditions. Additional negative drivers, including higher humidity (84%) and shortwave radiation (−1.7%), further support removal through dilution and oxidative processes.

Moderate positive impacts were also present. Latent heat flux (2.2%) and the 4-layer lifted index (1.6%) occasionally favored deeper boundary-layer development or transient vertical transport, permitting short-term accumulation of near-surface benzene. However, these effects remained secondary relative to the dominant negative contributions.

##### Setting C0—Warm-Season, High-Oxidant Dilution with Deep Mixing and Weak Combustion Inputs

Setting C0 emerged as another strong benzene-suppressing state, consistently associated with low concentrations (mean 0.44 µg m^−3^ and maximum 4.96 µg m^−3^) and limited internal variability. It reflects conditions dominated by weaker anthropogenic inputs and enhanced dispersion capacity and predominantly occurred from February through November, coinciding with moderate thermal conditions and stronger atmospheric dilution that reinforced its characterization as a dilution-dominated setting.

According to mean relative impacts (Table S8), the most influential suppressors are CO (−27%), latent heat flux (−6.9%), O_3_ (−5.5%), and NO_2_ (−3.2%). Mean CO concentrations were very low (0.21 mg m^−3^, occasionally up to 0.98 mg m^−3^), indicating reduced fossil-fuel combustion. Elevated O_3_ (mean 84 µg m^−3^ and maximum above 220 µg m^−3^) and moderate NO_2_ levels (about 13 µg m^−3^ and maximum 96 µg m^−3^) pointed to aged, well-ventilated air masses with strong oxidative removal rather than fresh urban emissions. The strong negative impact of CO was consistent with studies identifying it as a proxy for combustion emissions closely coupled with benzene under polluted conditions [43], where decreasing CO signals reduced the primary input and greater relative importance of atmospheric processing. A smaller but persistent negative contribution of SO_2_ (−2.4% impact), together with its low concentrations in this setting (mean 0.8 µg m^−3^, maximum 9 µg m^−3^) further supported the interpretation of limited industrial influence in C0.

Meteorological conditions supported this interpretation. Near-surface temperature and surface temperature, both with mean values around 20 °C but ranging from early-spring minima (around 7 °C) to midsummer highs above 34 °C, exerted strong negative contributions (−14.1% and −2.9%). These thermal conditions favored boundary-layer development, vertical mixing, and faster photochemical removal of benzene. Negative contributions of latent heat flux (−6.9%), combined with relatively large height with a boundary-layer (mean 848 m, occasionally exceeding 2600 m) and sensible heat flux (mean 292 W m^−2^), further confirmed the role of efficient turbulent mixing in diluting near-surface benzene [37].

##### Setting C3—Pre-COVID Summer Ventilation–Oxidation with Moderate Benzene and Strong Oxidizing Capacity

Setting C3 accounted for 5.2% of all observations and was characterized by mean benzene concentrations of 0.66 µg m^−3^ (maximum 5.44 µg m^−3^), indicating suppressive conditions, though weaker than in C0 and C1. It predominantly occurred from June to October, reflecting the summertime, but disappeared after October 2019, with no further occurrences during or after the COVID-19 period. This temporal pattern suggested that C3 was tied to pre-pandemic atmospheric and emission conditions, and its absence thereafter likely reflected broader shifts in structural anthropogenic activity.

Benzene suppression in this setting is primarily driven by negative contributions from planetary boundary-layer height (−0.7%), NO_2_ (−1.6%), O_3_ (−3.8%), surface temperature (−3.9%), latent heat flux (−5.8%), near-surface temperature (−15.6%), and CO (−19.2%) (Table S9). These drivers highlighted the role of dilution and oxidizing chemistry in maintaining low benzene concentrations. CO levels remained modest on average (0.26 mg m^−3^) but occasionally rose to 1.25 mg m^−3^. Their strong negative impact indicated that, in this setting, episodes with higher CO were nonetheless associated with cleaner states, likely to reflect pollution transport and efficient mixing rather than intense local emissions. Elevated ozone (mean 82 µg m^−3^, maximum 200 µg m^−3^) and moderate NO_2_ (16 µg m^−3^, maximum near 78 µg m^−3^) further supported oxidative removal under summer photochemical conditions.

Certain meteorological variables counterbalanced this suppression. Downward shortwave radiation (2.4%) enhanced surface heating and turbulence. With near-surface temperatures averaging 21 °C but reaching up to 37 °C, and boundary-layer heights typically 790 m but exceeding 2300 m in some cases, these factors can stimulate both vertical mixing and volatilization. This enables short-term increases in benzene levels despite the overall suppressive setting.

#### 3.2.3. Transitional Environmental Settings

Transitional ES capture intermediate atmospheric states that neither strongly enhance nor fully suppress benzene levels but instead reflect seasonal shifts and mixed source influences.

##### Setting C2—Transitional Accumulation with Moderate Benzene and SO_2_-Linked Diffuse Combustion Influence

Setting C2 accounted for a moderate share of the dataset (4.8%) and typically occurred between March and October, reflecting a transitional accumulation setting associated with generally moderate benzene levels and an absence of major pollution episodes (Figure S1). Mean benzene concentrations in C2 remained modest (0.63 µg m^−3^), though maximum values occasionally reached 3.67 µg m^−3^, indicating sporadic excursions above background conditions rather than sustained high-exposure events (Table S4).

The most prominent driver of benzene variability in this setting was SO_2_, with a positive mean normalized impact of 12% and excursions up to 31% (Table S10). Its average concentrations were relatively low (2.9 µg m^−3^) but can spike above 27 µg m^−3^ (Table S10), suggesting contributions from fossil-fuel combustion, likely industrial or diffuse urban sources rather than intensive residential heating [44]. However, given the moderate model performance (R^2^ = 0.56) and relatively high errors (Table S13), this association should be interpreted cautiously. Other pollutants, including CO (−22%), NO_2_ (−5.4%), and O_3_ (−4.3%), exerted negative mean impacts. Their mean levels were modest, about 0.2 mg m^−3^, 9.3 µg m^−3^, and 72 µg m^−3^, respectively. These negative contributions suggested that their variability does not reinforce benzene, and the weaker explanatory power pointed to possible noise in the model signal.

The meteorological profile of C2 aligned with weakly stable conditions that moderately constrained dispersion (Table S10). Boundary-layer heights were relatively shallow being on average 533 m (in an interval from 20 to 2429 m), relative humidity was elevated (68%, with episodes above 95%), and surface pressure was somewhat higher (1016 hPa), indicating anticyclonic influence. Near-surface temperatures averaged at 19 °C but ranged widely from early-spring minima (around 7 °C) to summer highs above 34 °C, reflecting the seasonal breadth of this setting. Low cloud cover (around 9%) further supported stable conditions, though not to the extent of triggering severe accumulation as in C6. Collectively, these features highlighted C2 as a transitional setting in which benzene concentrations remained moderate, shaped by a balance of diffuse combustion inputs and meteorological conditions that limited but did not prevent dispersion entirely.

##### Setting C5—Late-Winter-to-Spring Transition with Moderate Benzene Under Stable Anticyclonic Conditions

Setting C5 occurred predominantly during the late-winter-to-spring transition (February–May) and is characterized by relatively low-to-moderate benzene concentrations (mean 0.61 µg m^−3^, maximum 1.94 µg m^−3^, Table S4, Figure S1). This indicated that the setting was not associated with severe pollution episodes but rather reflected background-to-moderate conditions typical of seasonal change.

The setting was characterized by systematically negative contributions from several co-pollutants, most notably CO (−29%), NO_2_ (−7.2%), and O_3_ (−8.2%) (Table S11). Average levels of these pollutants remained modest, about 0.2 mg m^−3^, 7.9 µg m^−3^, and 77 µg m^−3^, respectively. However, occasional peaks were higher (CO up to 0.36 mg m^−3^, NO_2_ 47 µg m^−3^, O_3_ 170 µg m^−3^), highlighting that even under elevated levels, these pollutants were not associated with reinforcement of benzene. Instead, their negative impacts suggested processes that dilute or counterbalance benzene concentrations. SO_2_, with a mean value of 0.75 µg m^−3^ and maximum of 2 µg m^−3^, also contributed negatively (−3.0%), indicating that this setting is weakly associated with sulfur-containing combustion sources. Taken together, these findings pointed to a decoupling of benzene from both traffic-related and industrial heating emissions during the spring transition period.

Meteorological factors exerted a more nuanced influence (Table S11). Air temperature, with values ranging from −5.4 °C on cold late-winter days to over 10 °C in early spring, contributed positively (mean impact 9.2%) suggesting that rising temperatures promoted volatilization and sustained moderate benzene levels. The downward shortwave flux also showed a positive impact (3.1%), consistent with stronger insolation in this period. By contrast, boundary-layer height, with a mean of 878 m, had a negative impact (−1.1%) indicating that shallow-to-moderate mixing depths favored near-surface accumulation. Elevated relative humidity (mean 62%, with peaks above 95%), higher sea-level pressure (mean 1022 hPa), and relatively low cloud cover further supported stable, anticyclonic conditions that limited dispersion.

Collectively, these features defined C5 as a transitional setting in which benzene persisted at moderate levels. This persistence is not driven by strong emissions, but by seasonal conditions that restrict dispersion and allow short-term accumulation near the ground. As a result, C5 contrasts with the strongly polluted settings C6 and C4, where elevated benzene levels are primarily emission-driven.

## 4. Limitations

This study demonstrated the potential of an integrated, interpretable AI framework for analyzing benzene dynamics under diverse ES, yet several limitations should be acknowledged.

Model performance: Although the globally optimized ensemble achieved high overall performance (R^2^ = 0.87), as reflected by low aggregate errors (MAE = 0.23, RMSE = 0.44) and high explained variance (0.88), predictive accuracy, and hence explanatory power, varied across settings (Table S13). Clean and transitional settings, such as C0 and C2, showed lower explanatory power (R^2^ = 0.56–0.61), together with higher relative errors (MAPE up to 0.99), indicating that residual variability under low-pollution conditions was influenced by processes and micro-scale heterogeneity that are not fully captured by the available predictors. Intermediate and mixed regimes (C3–C5) showed more balanced performance, with consistently higher explained variance (0.61–0.86), lower absolute errors (MAE = 0.11–0.19), and reduced maximum errors, suggesting a more stable and interpretable model response. In contrast, pollution-enhancing settings, such as C6, combined the highest explained variance (R^2^ = 0.89) with larger absolute errors (MAE = 0.38; RMSE = 0.67; Max Error = 4.75), indicating that while the model robustly captures the overall structure and drivers of extreme benzene episodes, it still struggles to resolve the finest-scale temporal variability during peak events. In the absence of explicit prediction intervals or formal uncertainty bounds, these setting-dependent errors imply that SHAP-derived feature attributions should be interpreted as probabilistic, regime-level indicators of dominant controls rather than as deterministic, pointwise explanations. Overall, these performance patterns underscore the need for caution when interpreting results for cleaner or weakly structured regimes.

Predictor set and data coverage: The analysis relied on a limited set of five routinely monitored atmospheric pollutants (benzene, O_3_, CO, SO_2_, and NO_2_) and reanalysis-based meteorological parameters from a single urban background station (Zagreb, Croatia). While this reflected realistic monitoring constraints and highlighted the applicability of the framework under operational conditions, reliance on a single urban background site does not capture potential differences in benzene behavior across functional zones such as intensive traffic or industrial areas. Consequently, the generalization of the identified regimes should be interpreted within the boundaries of urban background conditions. Expanding the predictor set and incorporating multi-site or regional observations would likely enhance explanatory power, improve generalizability, and enable clearer discrimination of source- and setting-specific benzene pollution patterns. Recent studies have shown that integrating direct mobility and traffic indicators with air quality monitoring and advanced ML architectures can substantially enhance prediction accuracy for traffic-linked pollutants, highlighting a promising extension of the present framework where such data are available [45].

Clustering resolution: The setting-level clustering captured dominant patterns in benzene behavior and identified a certain number of influential ES. However, clustering resolution remains a constraint. While sub-clustering was performed for all settings (Table S12, Supplementary Materials), detailed analysis was presented only for dominant settings (C6 and C4) whereas systematic hierarchical treatment across all settings, explicitly incorporating SHAP interaction terms or season-specific projection, was beyond the scope of this study. Rare pollution extremes and atypical meteorological events were also underrepresented, further limiting assessment of high-impact but low-frequency conditions. In addition, a sizeable residual group (C-1) with heterogeneous impact signatures indicated that a fraction of observations cannot yet be assigned to physically coherent settings with high confidence; further investigation is therefore required to better understand its specific characteristics.

Future directions: Future work should broaden predictor coverage and refine setting identification. Systematic hierarchical sub-clustering of all settings, incorporating SHAP interaction terms, would allow a more granular characterization of regime heterogeneity beyond the dominant settings explored here. In addition, future work will examine the sensitivity of the SHAP-based embedding and clustering framework to key methodological choices, including dimensionality-reduction methods, clustering parameters, and random initialization, to further assess the robustness of the identified ES. Incorporating particulate matter, specified VOCs, oxidant proxies, land-use and mobility data, and detailed emission inventories would strengthen links between ES, sources, and exposure. Extending the framework to multi-station and multi-city datasets would allow evaluation of spatial robustness and transferability. Methodological advances such as hierarchical clustering, explicit use of SHAP interactions, and multi-output modeling (e.g., joint prediction of benzene, NO_2_, and O_3_) could better capture coupled pollutant behavior. Integrating interpretable AI with chemical transport models also offers strong potential for constraining extreme events, exploring emission scenarios, and enhancing policy-relevant insights.

## 5. Conclusions

This study applies an interpretable, setting-based AI framework to better understand atmospheric pollutant dynamics. Using seven years of hourly criteria pollutants and meteorological data from Zagreb (Croatia), we combined a metaheuristically optimized ensemble decision tree model with SHAP-based interpretation and density-based clustering. The resulting model accurately predicts benzene concentrations while enabling a process-oriented understanding of their variability. By clustering SHAP values rather than raw concentrations, we identified distinct ES, coherent multivariate states of meteorology and co-pollutants, that govern benzene behavior. This moves beyond conventional concentration-focused or source-apportionment approaches and frames benzene dynamics as emergent outcomes of ES in which meteorological conditions control the dispersion and accumulation of emitted pollutants rather than reflecting simple responses to type and strength of individual sources.

The results show that only a few ES-dominated benzene extremes. A winter stagnation setting (C6) and a pre-COVID multi-season accumulation setting (C4) emerged as the main pollution-enhancing settings. C6 was characterized by shallow boundary layers, weak winds, high humidity, and strong combustion influence, producing the highest benzene levels. C4 reflected weakly ventilated conditions with elevated heat fluxes and was present only before 2020, consistent with structural changes in anthropogenic activity. In contrast, warm-season dilution and oxidation settings (C0, C1, C3) were associated with low benzene concentrations, while C2 and C5 represented transitional settings with moderate levels. Overall, benzene extremes arise from a small number of distinct, physically interpretable settings rather than from continuous variability.

A key conceptual outcome of this work is that changes in the frequency and structure of ES provided a richer description of system evolution than trends in mean concentrations alone. The disappearance of C4 and C3 after 2020, the dominance of C6-like stagnation settings during early years, and the increasing prevalence of dilution/oxidation settings in later, cleaner intervals demonstrated that benzene dynamics respond to shifts in ES as much as to gradual emission reductions. This setting-aware perspective helped reconcile declining NO_2_ and CO, persistent O_3_ exceedances and episodic benzene peaks within a single coherent framework and emphasized that air quality management must account not only for emission magnitudes but also for the atmospheric contexts in which they operate.

This study demonstrated that interpretable, setting-based AI can deliver both strong predictive performance and meaningful physical insight into urban benzene dynamics. By identifying pollution-enhancing and pollution-suppressing ES, the framework provides a scalable basis for understanding exposure drivers and supporting targeted, context-aware mitigation strategies.

## Figures and Tables

**Table 1:** Statistical characteristics of the clusters.

Cluster	Number of Instances	Share of Instances [%]	Mean Benzene Level [µg m^−3^]	Mean Impact [µg m^−3^]	Mean Absolute Impact [µg m^−3^]	Mean Relative Impact [%]	Mean Absolute Normalized Impact [%]
C-1	2624	33.5	1.16	0.14	1.35	13.6	132.6
C0	1324	16.9	0.44	−0.58	1.01	−57.4	99.1
C1	690	8.8	0.3	−0.7	0.93	−68.7	92.0
C2	377	4.2	0.63	−0.39	1.06	−38.5	104.5
C3	409	5.2	0.66	−0.36	1.23	−35.1	120.6
C4	834	10.7	1.24	0.22	1.2	21.7	116.9
C5	243	3.1	0.6	−0.39	0.91	−38.0	89.7
C6	1324	16.9	2.02	0.99	2.07	97.7	203.3

## Data Availability

The data presented in this study are available on request from the corresponding author as they are being used in another ongoing research.

## Supplementary Materials

The following supporting information can be downloaded at: https://www.mdpi.com/article/10.3390/toxics14020181/s1, Table S1. Meteorological variables from the Global Data Assimilation System (GDAS1), operated by the National Oceanic and Atmospheric Administration (NOAA), U.S. Department of Commerce. Table S2. Descriptive statistics of air pollutant concentrations and meteorological parameters (2017–2023) at the Ksaverska cesta automatic monitoring station, Zagreb, Croatia. Table S3. Yearly descriptive statistics of pollutant concentrations (2017–2023) at the Ksaverska cesta automatic monitoring station, Zagreb, Croatia. Table S4. Benzene level within each cluster. Table S5. Predictor importance and characteristic environmental conditions for setting C6. Table S6. Predictor importance and characteristic environmental conditions for setting C4. Table S7. Predictor importance and characteristic environmental conditions for setting C1. Table S8. Predictor importance and characteristic environmental conditions for setting C0. Table S9. Predictor importance and characteristic environmental conditions for setting C3. Table S10. Predictor importance and characteristic environmental conditions for setting C2. Table S11. Predictor importance and characteristic environmental conditions for setting C5. Table S12. Distribution of benzene values and SHAP-based impact characteristics across subclusters within the identified settings. Table S13. Predictive performance of the Extra Trees model across atmospheric settings. Figure S1. Multi-scale temporal patterns of benzene concentrations [µg m^−3^].

## References

[1] World Health Organization (2025). WHO Strategic Approach on Air Quality, Energy Access and Health.

[2] Cohen A.J., Brauer M., Burnett R., Anderson H.R., Frostad J., Estep K., Balakrishnan K., Brunekreef B., Dandona L., Dandona R. (2017). Estimates and 25-year trends of the global burden of disease attributable to ambient air pollution: An analysis of data from the Global Burden of Diseases Study 2015. Lancet.

[3] Nie Y., Yan Y., Ji Y., Gao R., Ren Y., Bi F., Shang F., Li J., Chu W., Li H. (2025). Assessing the PM_2.5_–O_3_ Correlation and Unraveling Their Drivers in Urban Environment: Insights from the Bohai Bay Region, China. Atmosphere.

[4] Radić N., Perišić M., Jovanović G., Bezdan T., Stanišić S., Stanić N., Stojić A. (2025). An AI-based framework for characterizing the atmospheric fate of air pollutants within diverse environmental settings. Atmosphere.

[5] Ren Y., Wei J., Wang G., Wu Z., Ji Y., Li H. (2025). Significant contributions of biomass burning to PM2.5-bound aromatic compounds. Atmos. Chem. Phys..

[6] Li X.-B., Yuan B., Huangfu Y., Yang S., Song X., Qi J., He X., Wang S., Chen Y., Yang Q. (2025). Vertical changes in volatile organic compounds (VOCs) and impacts on photochemical ozone formation. Atmos. Chem. Phys..

[7] Ge Y., Solberg S., Heal M.R., Reimann S., van Caspel W., Hellack B., Salameh T., Simpson D. (2024). Evaluation of modelled versus observed non-methane volatile organic compounds at European Monitoring and Evaluation Programme sites in Europe. Atmos. Chem. Phys..

[8] Chadalavada S., Faust O., Salvi M., Seoni S., Raj N., Raghavendra U., Gudigar A., Barua P.D., Molinari F., Acharya R. (2025). Application of artificial intelligence in air pollution monitoring and forecasting: A systematic review. Environ. Model. Softw..

[9] Özüpak Y., Alpsalaz F., Aslan E. (2025). Air Quality Forecasting Using Machine Learning: Comparative Analysis and Ensemble Strategies for Enhanced Prediction. Water Air Soil Pollut..

[10] Reichstein M., Camps-Valls G., Stevens B., Jung M., Denzler J., Carvalhais N., Prabhat (2019). Deep learning and process understanding for data-driven Earth system science. Nature.

[11] Lundberg S.M., Lee S.I. (2017). A unified approach to interpreting model predictions. Adv. Neural Inf. Process. Syst..

[12] McInnes L., Healy J., Astels S. (2017). HDBSCAN: Hierarchical density-based clustering. J. Open Source Softw..

[13] Morales-García J., Ramos-Sorroche E., Balderas-Díaz S., Guerrero-Contreras G., Muñoz A., Santa J., Terroso-Sáenz F. (2025). Exploiting synthetic data generation to enhance pollution prediction. Appl. Soft Comput..

[14] (2012). Ambient Air—Standard Method for the Measurement of the Concentration of Nitrogen Dioxide and Nitrogen Monoxide by Chemiluminescence.

[15] (2012). Ambient Air—Standard Method for the Measurement of the Concentration of Sulphur Dioxide by Ultraviolet Fluorescence.

[16] (2012). Ambient Air—Standard Method for the Measurement of the Concentration of Ozone by Ultraviolet Photometry.

[17] (2012). Ambient Air—Standard Method for the Measurement of the Concentration of Carbon Monoxide by Non-Dispersive Infrared Spectroscopy.

[18] (2015). Ambient Air—Standard Method for the Measurement of Benzene Concentrations—Part 3: Automated Pumped Sampling with in Situ Gas Chromatography.

[19] (2017). General Requirements for the Competence of Testing and Calibration Laboratories. 3rd Edition.

[20] Dietterich T.G. (2000). Ensemble methods in machine learning. International Workshop on Multiple Classifier Systems.

[21] Python Software Foundation (2024). Python Language Reference.

[22] Wang Y., Huang H., Rudin C., Shaposhnik Y. (2021). Understanding how dimension reduction tools work: An empirical approach to deciphering t-SNE, UMAP, TriMap, and PaCMAP for data visualization. J. Mach. Learn. Res..

[23] R Core Team (2023). R: A Language and Environment for Statistical Computing.

[24] Geurts P., Ernst D., Wehenkel L. (2006). Extremely randomized trees. Mach. Learn..

[25] Pedregosa F., Varoquaux G., Gramfort A., Michel V., Thirion B., Grisel O., Blondel M., Prettenhofer P., Weiss R., Dubourg V. (2011). Scikit-learn: Machine learning in Python. J. Mach. Learn. Res..

[26] Ke G., Meng Q., Finley T., Wang T., Chen W., Ma W., Ye Q., Liu T.Y. (2017). LightGBM: A highly efficient gradient boosting decision tree. Adv. Neural Inf. Process. Syst..

[27] Chen T., Guestrin C. XGBoost: A Scalable Tree Boosting System. Proceedings of the 22nd ACM SIGKDD International Conference on Knowledge Discovery and Data Mining (KDD’16).

[28] Heidari A.A., Mirjalili S., Faris H., Aljarah I., Mafarja M., Chen H. (2019). Harris hawks optimization: Algorithm and applications. FGCS.

[29] Mirjalili S. (2016). SCA: A sine cosine algorithm for solving optimization problems. Knowl.-Based Syst..

[30] Stojić A., Jovanović G., Stanišić S., Romanić S.H., Šoštarić A., Udovičić V., Perišić M., Milićević T. (2022). The PM2.5-bound polycyclic aromatic hydrocarbon behavior in indoor and outdoor environments, part II: Explainable prediction of benzo [a] pyrene levels. Chemosphere.

[31] Jakovljević I., Sever Štrukil Z., Godec R., Davila S., Pehnec G. (2021). Influence of lockdown caused by the COVID-19 pandemic on air pollution and carcinogenic content of particulate matter observed in Croatia. Air Qual. Atmos. Health.

[32] Lovrić M., Antunović M., Šunić I., Vuković M., Kecorius S., Kröll M., Bešlić I., Godec R., Pehnec G., Geiger B. (2022). Machine learning and meteorological normalization for assessment of particulate matter changes during the COVID-19 lockdown in Zagreb, Croatia. Int. J. Environ. Res. Public Health.

[33] Copernicus Atmosphere Monitoring Service (CAMS) (2022). Repeated Summer Heatwaves Trigger New Ozone-Level Peaks Throughout Europe. https://atmosphere.copernicus.eu/repeated-summer-heatwaves-trigger-new-ozone-level-peaks-throughout-europe.

[34] Matasović B., Pehnec G., Bešlić I., Babić D. (2021). Assessment of ozone concentration data from the northern Zagreb area, Croatia, for the period from 2003 to 2016. Environ. Sci. Pollut. Res..

[35] Whaley C.H., Galarneau E., Makar P.A., Moran M.D., Zhang J. (2020). How much does traffic contribute to benzene and polycyclic aromatic hydrocarbon air pollution? Results from a high-resolution North American air quality model centred on Toronto, Canada. Atmos. Chem. Phys..

[36] Yuval Levi Y., Dayan U., Levy I., Broday M.D. (2020). On the association between characteristics of the atmospheric boundary layer and air pollution concentrations. Atmos. Res..

[37] Wang L., Wang H., Liu J., Gao Z., Yang Y., Zhang X., Li Y., Huang M. (2019). Impacts of the near-surface urban boundary layer structure on PM2.5 concentrations in Beijing during winter. Sci. Total Environ..

[38] Calvert J.G., Atkinson R., Becker K.H., Kamens R.M., Seinfeld J.H., Wallington T.J., Yarwood G. (2002). Aerosol generation in atmospheric oxidation of aromatic hydrocarbons. The mechanisms of Atmospheric Oxidation of Aromatic Hydrocarbons.

[39] George C., Ammann M., D’Anna B., Donaldson D.J., Nizkorodov S.A. (2015). Heterogeneous photochemistry in the atmosphere. Chem. Rev..

[40] Seinfeld J.H., Pandis S.N. (2016). Atmospheric Chemistry and Physics: From Air Pollution to Climate Change.

[41] Von Schneidemesser E., Monks S.P., Allan J.D., Bruhwiler L., Forster P., Fowler D., Lauer A., Morgan T.W., Paasonen P., Righi M. (2015). Chemistry and the Linkages between Air Quality and Climate Change. Chem. Rev..

[42] Song X., Li X.B., Yuan B., He X., Chen Y., Wang S., Huangfu Y., Peng Y., Zhang C., Liu A. (2024). Elucidating key factors in regulating budgets of ozone and its precursors in atmospheric boundary layer. npj Clim. Atmos. Sci..

[43] Sanda M., Dunea D., Iordache S., Pohoata A., Glod-Lendvai A.-M., Onutu I. (2023). A three-year analysis of toxic benzene levels and associated impact in Ploieşti city, Romania. Toxics.

[44] Ahmed M., Rappenglück B., Das S., Chellam S. (2021). Source apportionment of volatile organic compounds, CO, SO_2_ and trace metals in a complex urban atmosphere. Environ. Adv..

[45] Morales-García J., Ramos-Sorroche E., Balderas-Díaz S., Guerrero-Contreras G., Muñoz A., Santa J., Terroso-Sáenz F. (2025). Reducing Pollution Health Impact with Air Quality Prediction Assisted by Mobility Data. IEEE J. Biomed. Health Inform..

