# Supplementary material for: Uncovering Benzene Pollution Patterns Using an Interpretable, Setting-Aware Artificial Intelligence Approach
Source: Toxics. 2026 Feb 18;14(2):181. doi: 10.3390/toxics14020181 (PMC12944871; doi:10.3390/toxics14020181)
Supplement: Supplementary file 1 [file toxics-14-00181-s001.zip › toxics-4103947-supplementary.pdf]

Uncovering benzene pollution patterns using an interpretable, setting-aware artificial intelligence approach

[1] Institute for Medical Research and Occupational Health, Ksaverska cesta 2, PO Box 291, 10001 Zagreb, Croatia; ibeslic@imi.hr; sdavila@imi.hr; gpehnec@imi.hr; sherceg@imi.hr
[2] Faculty of Informatics and Computing, Singidunum University, Danijelova 32, 11000 Belgrade, Serbia; tbezdan@singidunum.ac.rs
[3] Institute of Physics Belgrade, a National Institute of the Republic of Serbia, Pregrevica 118, 11080 Belgrade, Serbia; gordana.jovanovic@ipb.ac.rs; andreja.stojic@ipb.ac.rs; mirjana.perisic@ipb.ac.rs
[4] Environment and Sustainable Development Studies, Singidunum University, Danijelova 32, 11000 Belgrade, Serbia

- Correspondence: gordana.jovanovic@ipb.ac.rs

Supplementary material

Table S1. Meteorological variables from the Global Data Assimilation System (GDAS1), operated by National Oceanic and Atmospheric Administration (NOAA), U.S. Department of Commerce.

Field Description	*Units*	*Label*
Pressure at surface	*hPa*	*prss*
Pressure reduced to mean sea level	*hPa*	*mslp*
Accumulated precipitation (6-hour accumulation)	*m*	*tpp6*
Momentum flux intensity (3- or 6-hour average)	$N\ m^{-2}$	*mofi*
Momentum flux direction (3- or 6-hour average)	$N\ m^{-2}$	*mofd*
Sensible heat net flux at surface (3- or 6-hour average)	$W\ m^{-2}$	*shif*
Downward short-wave radiation flux (3- or 6-hour average)	$W\ m^{-2}$	*dswf*
Relative humidity at 2 m above ground level (AGL)	%	*rh2m*
Wind direction at 10 m AGL	$m\ s^{-1}$	*wd10m*
Wind speed at 10 m AGL	$m\ s^{-1}$	*ws10m*
Temperature at 2 m AGL	*K*	*t02m*
Total cloud cover (3- or 6-hour average)	%	*tcld*
Convective available potential energy	$J\ kg^{-1}$	*cape*
Convective inhibition	$J\ kg^{-1}$	*cinh*
Standard lifted index	*K*	*lisd*
Best 4-layer lifted index	*K*	*lib4*
Planetary boundary layer height	*m*	*pblh*
Temperature at surface	*K*	*tmps*
Accumulated convective precipitation (6-hour accumulation)	*m*	*cpp6*
Volumetric soil moisture content	*fraction*	*solm*
Categorical snow (yes = 1, no = 0; 3- or 6-hour average)	/	*csno*
Categorical ice (yes = 1, no = 0; 3- or 6-hour average)	/	*cice*
Categorical freezing rain (yes = 1, no = 0; 3- or 6-hour average)	/	*cfzr*
Categorical rain (yes = 1, no = 0; 3- or 6-hour average)	/	*crai*
Latent heat net flux at surface (3- or 6-hour average)	$W\ m^{-2}$	*lhtf*
Low cloud cover (3- or 6-hour average)	%	*lcld*
Middle cloud cover (3- or 6-hour average)	%	*mcld*
High cloud cover (3- or 6-hour average)	%	*hcld*

Table S2. Descriptive statistics of air pollutant concentrations and meteorological parameters (2017–2023) at the Ksaverska cesta automatic monitoring station, Zagreb, Croatia.

Variable	*mean*	*std*	*min*	*25%*	*50%*	*75%*	*max*
NO_2 [μg m^{-3}]	*16.8*	*14.6*	*0.00*	*5.9*	*11.9*	*23.8*	*126.5*
CO [mg m^{-3}]	*0.32*	*0.23*	*0.00*	*0.19*	*0.24*	*0.37*	*4.09*
SO_2 [μg m^{-3}]	*1.3*	*2.1*	*0.00*	*0.5*	*0.9*	*1.5*	*84.9*
O_3 [μg m^{-3}]	*54.1*	*37.9*	*0.00*	*22.8*	*50.2*	*78.0*	*249.9*
Benzene [μg m^{-3}]	*1.02*	*1.20*	*0.00*	*0.32*	*0.60*	*1.27*	*13.52*
prss	*985.26*	*7.46*	*955.10*	*980.81*	*985.30*	*989.91*	*1014.35*
mslp	*1017.66*	*7.80*	*985.61*	*1012.94*	*1017.42*	*1022.36*	*1047.76*
tpp6	*0.00*	*0.00*	*0.00*	*0.00*	*0.00*	*0.00*	*0.02*
shif	*16.71*	*61.42*	*-168.01*	*-24.41*	*-4.07*	*39.63*	*301.47*
dswf	*168.35*	*213.51*	*0.00*	*1.33*	*61.28*	*283.36*	*792.06*
rh2m	*75.31*	*16.58*	*18.01*	*64.16*	*78.29*	*89.31*	*100.00*
wd10m	*164.80*	*88.26*	*0.38*	*78.61*	*177.89*	*232.40*	*357.64*
ws10m	*1.87*	*1.20*	*0.05*	*1.08*	*1.53*	*2.28*	*10.90*
t02m	*11.27*	*8.52*	*-14.99*	*4.74*	*10.81*	*17.40*	*36.86*
tcld	*43.08*	*37.24*	*0.00*	*4.71*	*33.33*	*79.94*	*100.00*
cape	*77.24*	*253.74*	*0.00*	*0.00*	*0.00*	*11.53*	*3707.27*
cinh	*0.05*	*0.10*	*0.00*	*0.00*	*0.00*	*0.05*	*0.93*
lisd	*7.56*	*6.68*	*-10.09*	*2.51*	*6.80*	*12.13*	*31.13*
lib4	*-268.23*	*5.02*	*-282.15*	*-271.93*	*-268.86*	*-265.00*	*-246.68*
pblh	*421.55*	*495.75*	*20.00*	*35.03*	*202.31*	*664.76*	*3587.51*
tmps	*11.13*	*8.90*	*-16.00*	*4.32*	*10.53*	*17.29*	*38.77*
cpp6	*0.00*	*0.00*	*0.00*	*0.00*	*0.00*	*0.00*	*0.01*
solm	*0.20*	*0.12*	*0.00*	*0.00*	*0.25*	*0.28*	*0.46*
csno	*0.02*	*0.12*	*0.00*	*0.00*	*0.00*	*0.00*	*1.00*
cice	*0.00*	*0.00*	*0.00*	*0.00*	*0.00*	*0.00*	*0.00*
cfzr	*0.00*	*0.00*	*0.00*	*0.00*	*0.00*	*0.00*	*0.00*
crai	*0.06*	*0.22*	*0.00*	*0.00*	*0.00*	*0.00*	*1.00*
lcld	*22.16*	*31.57*	*0.00*	*0.00*	*3.38*	*33.33*	*100.00*
lhtf	*39.36*	*38.15*	*0.00*	*6.49*	*22.44*	*75.69*	*100.00*
mcld	*19.44*	*30.27*	*0.00*	*0.00*	*0.88*	*30.50*	*100.00*
hcld	*29.60*	*34.50*	*0.00*	*0.02*	*12.30*	*57.73*	*100.00*
mofi	*0.12*	*0.17*	*0.00*	*0.03*	*0.06*	*0.13*	*2.32*
mofd	*165.05*	*88.12*	*0.00*	*78.91*	*181.45*	*226.02*	*359.81*

Table S3. Yearly descriptive statistics of pollutant concentrations (2017–2023) at the Ksaverska cesta automatic monitoring station, Zagreb, Croatia.

***Benzene** [μg m^{-3}]*								
	count	*mean*	*std*	*min*	*25%*	*50%*	*75%*	*max*
2017	*7068*	*1.18*	*1.46*	*0.00*	*0.34*	*0.64*	*1.52*	*13.45*

2018	*3856*	*1.50*	*1.46*	*0.01*	*0.47*	*1.01*	*2.06*	*13.52*
2019	*6594*	*0.98*	*1.05*	*0.01*	*0.33*	*0.59*	*1.23*	*13.51*
2020	*4459*	*0.97*	*1.27*	*0.00*	*0.31*	*0.53*	*1.05*	*12.25*
2021	*5420*	*0.98*	*1.02*	*0.01*	*0.32*	*0.58*	*1.27*	*8.35*
2022	*5780*	*0.63*	*0.77*	*0.00*	*0.13*	*0.39*	*0.83*	*8.32*
2023	*5947*	*1.04*	*1.15*	*0.01*	*0.42*	*0.69*	*1.20*	*9.98*
NO_2 *[μg m^{-3}]*								
	count	*mean*	*std*	*min*	*25%*	*50%*	*75%*	*max*
2017	*7068*	*18.7*	*15.7*	*0.1*	*6.8*	*13.7*	*26.5*	*126.5*
2018	*3856*	*20.2*	*16.1*	*0.0*	*7.5*	*14.9*	*29.5*	*94.9*
2019	*6594*	*17.1*	*15.1*	*0.0*	*5.8*	*11.7*	*24.8*	*101.8*
2020	*4459*	*16.4*	*14.7*	*0.0*	*4.8*	*11.7*	*24.2*	*96.1*
2021	*5420*	*16.4*	*12.8*	*0.3*	*6.6*	*12.4*	*22.9*	*94.8*
2022	*5780*	*15.2*	*13.3*	*0.00*	*5.5*	*11.0*	*21.0*	*99.4*
2023	*5947*	*14.3*	*13.3*	*0.00*	*4.7*	*9.6*	*19.6*	*96.0*
CO *[mg m^{-3}]*								
	count	*mean*	*std*	*min*	*25%*	*50%*	*75%*	*max*
2017	*7068*	*0.36*	*0.29*	*0.03*	*0.18*	*0.25*	*0.42*	*4.09*
2018	*3856*	*0.39*	*0.26*	*0.09*	*0.22*	*0.31*	*0.48*	*2.27*
2019	*6594*	*0.30*	*0.20*	*0.05*	*0.18*	*0.23*	*0.34*	*2.98*
2020	*4459*	*0.30*	*0.22*	*0.09*	*0.17*	*0.21*	*0.34*	*2.17*
2021	*5420*	*0.31*	*0.19*	*0.11*	*0.20*	*0.24*	*0.36*	*2.42*
2022	*5780*	*0.34*	*0.21*	*0.00*	*0.17*	*0.26*	*0.44*	*1.85*
2023	*5947*	*0.28*	*0.18*	*0.09*	*0.19*	*0.23*	*0.29*	*2.60*
SO_2 *[μg m^{-3}]*								
	count	*mean*	*std*	*min*	*25%*	*50%*	*75%*	*max*
2017	*7068*	*2.1*	*3.3*	*0.0*	*1.0*	*1.4*	*1.9*	*84.9*
2018	*3856*	*2.1*	*2.7*	*0.1*	*0.8*	*1.3*	*2.8*	*56.5*
2019	*6594*	*1.0*	*1.4*	*0.0*	*0.4*	*0.7*	*1.2*	*27.0*
2020	*4459*	*1.0*	*1.3*	*0.0*	*0.4*	*0.7*	*1.2*	*29.8*
2021	*5420*	*1.0*	*0.9*	*0.0*	*0.5*	*0.8*	*1.3*	*14.2*
2022	*5780*	*0.6*	*1.0*	*0.0*	*0.2*	*0.4*	*0.7*	*19.8*
2023	*5947*	*1.3*	*1.9*	*0.0*	*0.4*	*0.9*	*1.6*	*57.5*
O_3 *[μg m^{-3}]*								
	count	*mean*	*std*	*min*	*25%*	*50%*	*75%*	*max*
2017	*7068*	*53.8*	*36.7*	*0.0*	*25.2*	*49.7*	*77.0*	*206.4*
2018	*3856*	*46.5*	*36.1*	*0.0*	*15.9*	*41.0*	*67.6*	*179.4*
2019	*6594*	*55.2*	*38.4*	*0.0*	*23.5*	*50.4*	*80.9*	*249.9*
2020	*4459*	*52.1*	*33.1*	*0.0*	*23.0*	*53.7*	*75.7*	*169.6*
2021	*5420*	*61.6*	*39.8*	*0.1*	*28.1*	*59.9*	*90.7*	*232.4*
2022	*5780*	*59.7*	*44.6*	*0.0*	*21.1*	*54.0*	*90.9*	*230.3*
2023	*5947*	*47.2*	*31.6*	*0.0*	*22.5*	*44.2*	*66.6*	*179.3*

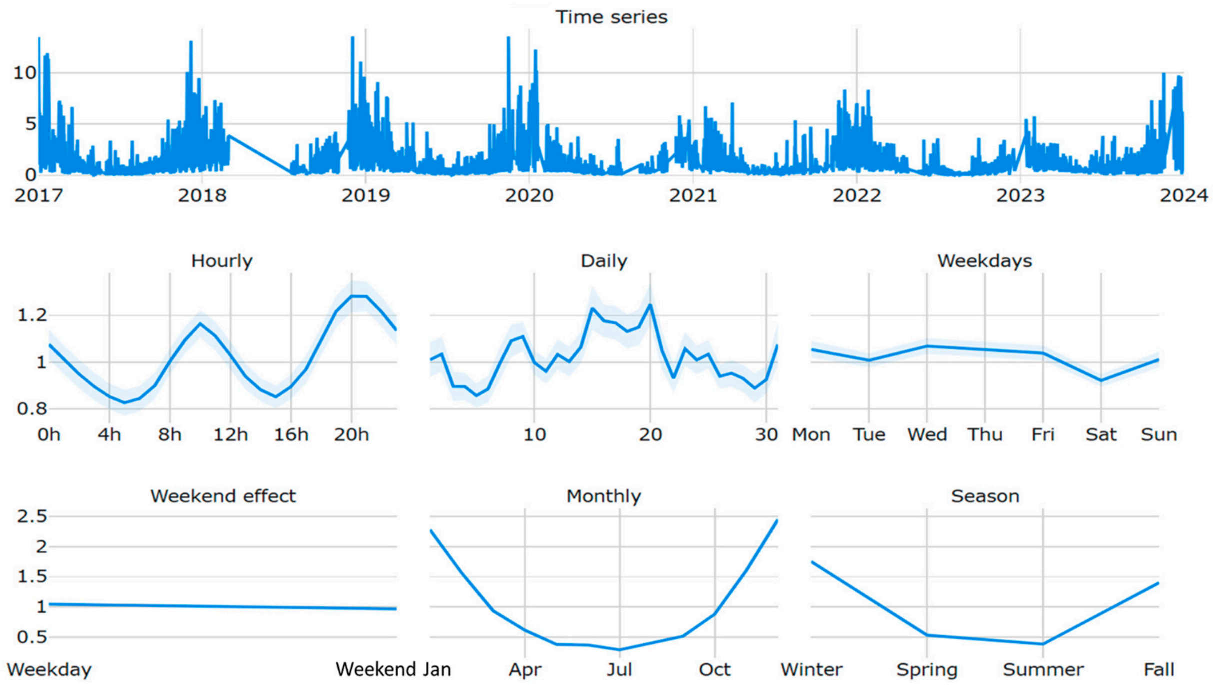

Figure S1. Multi-scale temporal patterns of benzene concentrations [μg m^{-3}].

Table S4. Benzene level within each cluster.

Cluster	*Minimum benzene concentrations [μg m^{-3}]*	*Mean benzene concentrations [μg m^{-3}]*	*Maximum benzene concentrations [μg m^{-3}]*
C0	*0.00*	*0.44*	*4.96*
C1	*0.01*	*0.30*	*1.44*
C2	*0.04*	*0.63*	*3.67*
C3	*0.02*	*0.66*	*5.44*
C4	*0.01*	*1.24*	*5.13*
C5	*0.02*	*0.61*	*1.94*
C6	*0.03*	*2.02*	*12.96*

Table S5. Predictor importance and characteristic environmental conditions for setting C6.

Variable	*Relative impact minimum [%]*	*Relative impact maximum [%]*	*Relative impact mean [%]*	*Minimum level [variables unit]*	*Maximum level [variables unit]*	*Mean level [variables unit]*
Benzene [μg m^{-3}]				*0.03*	*12.96*	*2.02*
CO [mg m^{-3}]	*-59.16*	*72.65*	*11.89*	*0.10*	*2.87*	*0.50*
t02m	*-16.87*	*26.98*	*7.14*	*-14.06*	*16.19*	*3.53*
lhtf	*-6.94*	*13.86*	*2.17*	*0.00*	*86.70*	*9.51*

tmps	-11.37	19.49	0.99	-16.00	15.94	2.88
lisd	-15.40	19.70	0.84	0.98	29.78	12.92
solm	-22.41	17.16	0.66	0.00	0.44	0.25
O_3 [μg m^{-3}]	-20.20	27.40	0.52	0.00	137.49	30.41
cape	-3.20	4.91	0.13	0.00	69.07	0.71
lcld	-6.85	7.64	0.12	0.00	100.00	26.92
mofi	-6.61	17.78	0.12	0.00	1.39	0.08
prss	-7.26	9.38	-0.18	956.51	1014.35	986.57
mofd	-5.87	11.22	-0.22	2.40	354.42	160.53
ws10m	-8.18	10.56	-0.24	0.20	7.44	1.58
mslp	-8.48	13.84	-0.25	988.62	1047.02	1019.62
rh2m	-12.44	5.56	-0.26	39.64	100.00	84.55
shif	-8.00	8.39	-0.47	-105.57	71.82	-16.57
lib4	-17.20	15.54	-0.49	-274.50	-250.71	-265.24
NO_2 [μg m^{-3}]	-28.20	23.35	-1.18	0.00	95.24	22.62
dswf	-7.60	7.84	-1.83	0.00	290.46	23.12
SO_2 [μg m^{-3}]	-19.40	20.21	-2.57	0.00	19.46	1.07

Table S6. Predictor importance and characteristic environmental conditions for setting C4.

Variable	*Relative impact minimum [%]*	*Relative impact maximum [%]*	*Relative impact mean [%]*	*Minimum level [variables unit]*	*Maximum level [variables unit]*	*Mean level [variables unit]*
Benzene [μg m^{-3}]				0.01	5.13	1.24
lhtf	-10.81	10.72	1.66	0.00	87.60	12.52
t02m	-28.85	29.05	1.56	-10.43	23.82	7.90
CO [mg m^{-3}]	-56.80	68.84	1.56	0.10	0.91	0.34
lib4	-16.06	9.96	0.96	-279.00	-252.40	-268.14
mcld	-2.97	4.94	0.28	0.00	100.00	26.63
hcld	-6.05	6.75	0.21	0.00	100.00	38.96
SO_2 [μg m^{-3}]	-14.38	15.78	0.19	0.01	28.63	1.27
prss	-4.52	5.96	0.17	957.41	1005.03	984.83
cinh	-4.47	3.92	0.14	0.00	0.50	0.05
mofi	-5.17	9.67	-0.10	0.00	1.30	0.08
O_3 [μg m^{-3}]	-11.65	16.55	-0.17	0.15	154.46	36.90
ws10m	-4.95	6.50	-0.22	0.22	7.21	1.55
wd10m	-9.54	7.97	-0.23	6.33	356.06	185.30
rh2m	-9.60	6.78	-0.29	42.21	99.22	82.78
tcld	-6.01	7.67	-0.29	0.00	100.00	56.82
NO_2 [μg m^{-3}]	-16.92	24.55	-0.38	0.03	74.68	19.73
mslp	-6.28	9.63	-0.63	988.89	1040.20	1017.93
tmps	-14.85	19.76	-0.69	-11.03	24.54	7.32
mofd	-6.61	10.13	-0.77	4.90	359.40	149.22
shif	-8.92	6.03	-0.90	-148.40	91.84	-19.67

lisd	-12.90	11.52	-1.02	-2.08	26.87	8.81
solm	-12.83	8.68	-1.39	0.00	0.38	0.00
dswf	-7.26	11.43	-1.74	0.00	352.12	30.21

Table S7. Predictor importance and characteristic environmental conditions for setting C1.

Variable	*Relative impact minimum [%]*	*Relative impact maximum [%]*	*Relative impact mean [%]*	*Minimum level [variables unit]*	*Maximum level [variables unit]*	*Mean level [variables unit]*
Benzene [μg m^{-3}]				0.01	1.44	0.30
lhtf	-2.93	7.98	2.16	0.00	61.60	11.09
lib4	-6.70	12.56	1.59	-280.51	-259.65	-271.44
pblh	-4.52	7.59	0.46	20.00	1196.98	108.95
prss	-8.24	5.74	0.42	969.54	1000.35	984.61
lcld	-4.45	5.48	0.39	0.00	99.98	12.32
mofi	-2.83	6.01	0.25	0.00	0.80	0.04
tcld	-4.16	4.14	-0.14	0.00	100.00	31.31
wd10m	-4.34	5.62	-0.18	8.81	354.70	206.81
hcld	-3.89	6.34	-0.18	0.00	100.00	23.70
ws10m	-5.90	4.56	-0.41	0.16	5.64	1.31
mslp	-4.79	4.92	-0.44	1001.23	1031.53	1016.14
shif	-6.69	5.53	-0.56	-97.30	23.51	-27.36
rh2m	-11.10	7.05	-0.63	50.91	99.56	83.57
mofd	-5.63	7.02	-0.98	4.95	352.16	155.72
dswf	-6.17	3.34	-1.74	0.00	156.85	17.17
solm	-19.67	10.34	-1.88	0.00	0.34	0.24
tmps	-10.55	2.46	-2.83	7.11	24.16	15.15
lisd	-11.30	5.55	-3.36	-5.89	16.51	4.17
SO_2 [μg m^{-3}]	-15.50	5.21	-4.07	0.00	1.99	0.59
O_3 [μg m^{-3}]	-15.57	16.20	-4.21	2.51	222.59	57.55
NO_2 [μg m^{-3}]	-17.94	8.88	-6.41	0.02	35.32	8.11
t02m	-25.62	-2.47	-12.67	8.00	24.13	15.75
CO [mg m^{-3}]	-58.57	14.32	-36.90	0.11	0.32	0.17

Table S8. Predictor importance and characteristic environmental conditions for setting C0.

Variable	*Relative impact minimum [%]*	*Relative impact maximum [%]*	*Relative impact mean [%]*	*Minimum level [variables unit]*	*Maximum level [variables unit]*	*Mean level [variables unit]*
Benzene [μg m^{-3}]				0.00	4.96	0.44
dswf	-2.91	10.83	2.34	0.00	770.62	443.37
shif	-5.89	9.03	2.23	-67.01	282.57	85.88

rh2m	*-6.35*	*10.89*	*1.39*	*22.56*	*98.45*	*60.81*
lib4	*-8.76*	*9.06*	*1.18*	*-280.61*	*-254.38*	*-270.78*
prss	*-6.99*	*8.33*	*0.33*	*958.75*	*1002.85*	*984.84*
mcld	*-2.71*	*4.77*	*0.26*	*0.00*	*99.98*	*11.23*
wd10m	*-5.65*	*6.61*	*0.25*	*7.31*	*347.44*	*148.51*
ws10m	*-5.18*	*5.94*	*0.22*	*0.18*	*8.23*	*2.14*
mofd	*-4.70*	*9.60*	*-0.15*	*8.57*	*348.91*	*170.38*
cape	*-5.30*	*5.55*	*-0.18*	*0.00*	*2928.17*	*209.85*
tcld	*-3.52*	*3.59*	*-0.22*	*0.00*	*100.00*	*34.44*
mofi	*-4.41*	*13.69*	*-0.41*	*0.00*	*1.57*	*0.15*
mslp	*-6.88*	*10.79*	*-0.52*	*990.45*	*1034.97*	*1016.36*
solm	*-13.35*	*7.70*	*-0.61*	*0.00*	*0.37*	*0.24*
pblh	*-9.21*	*9.32*	*-1.12*	*20.00*	*2684.14*	*848.49*
SO_2 [μg m^{-3}]	*-14.68*	*14.18*	*-2.36*	*0.00*	*9.10*	*0.80*
lisd	*-11.63*	*4.02*	*-2.83*	*-8.12*	*18.90*	*2.47*
tmps	*-13.58*	*3.41*	*-2.88*	*6.47*	*37.51*	*20.72*
NO_2 [μg m^{-3}]	*-20.36*	*35.01*	*-3.18*	*0.01*	*96.01*	*13.19*
O_3 [μg m^{-3}]	*-16.62*	*12.16*	*-5.49*	*0.21*	*224.85*	*84.42*
lhtf	*-15.75*	*2.14*	*-6.85*	*6.14*	*100.00*	*88.51*
t02m	*-32.78*	*9.08*	*-14.13*	*6.93*	*34.52*	*20.13*
CO [mg m^{-3}]	*-53.72*	*50.05*	*-26.65*	*0.03*	*0.98*	*0.21*

Table S9. Predictor importance and characteristic environmental conditions for setting C3.

Variable	*Relative impact minimum [%]*	*Relative impact maximum [%]*	*Relative impact mean [%]*	*Minimum level [variables unit]*	*Maximum level [variables unit]*	*Mean level [variables unit]*
Benzene [μg m^{-3}]				*0.02*	*5.44*	*0.66*
dswf	*-1.84*	*7.87*	*2.42*	*69.00*	*752.15*	*438.64*
shif	*-3.60*	*7.23*	*2.27*	*-36.18*	*225.18*	*81.88*
SO_2 [μg m^{-3}]	*-7.72*	*13.17*	*1.42*	*0.13*	*9.22*	*1.71*
lib4	*-2.99*	*10.85*	*1.39*	*-280.65*	*-261.41*	*-272.02*
rh2m	*-3.53*	*7.54*	*1.13*	*28.11*	*93.13*	*59.22*
prss	*-2.18*	*7.58*	*0.41*	*967.81*	*996.92*	*983.92*
wd10m	*-4.47*	*6.35*	*0.29*	*7.44*	*344.07*	*148.79*
mofd	*-3.45*	*8.99*	*0.22*	*5.96*	*339.06*	*171.04*
mcld	*-2.33*	*3.86*	*0.12*	*0.00*	*99.84*	*10.94*
hcld	*-3.53*	*6.26*	*0.11*	*0.00*	*99.84*	*30.54*
lcld	*-2.29*	*4.48*	*0.11*	*0.00*	*99.35*	*11.07*
cape	*-4.42*	*3.14*	*-0.28*	*0.00*	*2461.48*	*247.67*
mofi	*-4.90*	*9.14*	*-0.31*	*0.01*	*1.15*	*0.15*

mslp	-4.57	7.68	-0.64	1000.26	1029.41	1016.11
pblh	-7.64	4.51	-0.73	20.00	2329.59	790.49
NO_2 [μg m^{-3}]	-14.91	22.32	-1.60	0.44	78.00	16.19
solm	-9.41	3.89	-1.65	0.00	0.00	0.00
lisd	-6.38	4.31	-1.81	-8.13	14.32	1.37
O_3 [μg m^{-3}]	-11.64	10.48	-3.78	1.60	199.75	81.93
tmps	-12.69	0.58	-3.85	7.93	37.17	21.56
lhtf	-13.27	1.06	-5.82	29.36	100.00	88.43
t02m	-35.69	2.01	-15.59	9.18	36.86	21.07
CO [mg m^{-3}]	-53.10	53.64	-19.24	0.09	1.25	0.26

Table S10. Predictor importance and characteristic environmental conditions for setting C2.

Variable	*Relative impact minimum [%]*	*Relative impact maximum [%]*	*Relative impact mean [%]*	*Minimum level [variables unit]*	*Maximum level [variables unit]*	*Mean level [variables unit]*
Benzene [μg m^{-3}]				0.04	3.67	0.63
SO_2 [μg m^{-3}]	0.89	31.35	11.96	1.34	27.44	2.89
lib4	-9.26	17.11	1.17	-279.67	-259.19	-270.75
shif	-9.50	6.54	0.84	-69.30	247.11	34.24
lcld	-7.34	5.04	0.56	0.00	100.00	9.33
rh2m	-8.57	9.02	0.40	21.36	98.79	68.04
prss	-5.89	7.83	0.36	967.03	1001.36	984.98
ws10m	-3.93	5.92	0.15	0.36	5.74	1.64
mcld	-2.74	5.33	0.15	0.00	99.84	9.95
mslp	-6.44	9.68	-0.10	998.14	1031.09	1016.43
mofd	-7.62	11.99	-0.14	8.61	352.94	175.92
hcld	-4.47	5.15	-0.21	0.00	99.51	21.45
pblh	-6.62	9.19	-0.26	20.00	2428.78	533.59
dswf	-5.32	5.59	-0.26	0.00	769.32	242.21
solm	-11.34	21.18	-0.59	0.00	0.36	0.22
lisd	-11.79	4.79	-2.07	-7.40	16.95	3.86
lhtf	-14.22	7.54	-2.61	0.00	100.00	50.52
tmps	-12.74	5.54	-2.66	6.82	36.07	18.70
O_3 [μg m^{-3}]	-14.14	22.95	-4.27	0.55	204.68	72.16
NO_2 [μg m^{-3}]	-14.85	8.29	-5.44	0.05	39.44	9.34
t02m	-30.86	7.57	-13.56	7.31	34.69	18.73
CO [mg m^{-3}]	-49.84	26.83	-22.06	0.11	0.65	0.22

Table S11. Predictor importance and characteristic environmental conditions for setting C5.

Variable	*Relative impact minimum [%]*	*Relative impact maximum [%]*	*Relative impact mean [%]*	*Minimum level [variables unit]*	*Maximum level [variables unit]*	*Mean level [variables unit]*
Benzene [μg m^{-3}]				*0.02*	*1.94*	*0.61*
t02m	*-1.64*	*24.95*	*9.19*	*-5.41*	*10.37*	*5.20*
dswf	*-1.09*	*8.05*	*3.12*	*0.00*	*630.28*	*271.24*
shif	*-3.61*	*10.78*	*2.27*	*-61.94*	*253.39*	*53.91*
rh2m	*-5.24*	*7.48*	*1.83*	*24.13*	*96.50*	*61.72*
ws10m	*-3.36*	*5.74*	*1.09*	*0.74*	*8.63*	*3.05*
solm	*-8.94*	*9.02*	*0.73*	*0.00*	*0.38*	*0.24*
wd10m	*-3.02*	*4.02*	*0.65*	*10.26*	*353.23*	*94.82*
lisd	*-7.60*	*12.55*	*0.34*	*-0.23*	*24.00*	*11.44*
mofd	*-4.10*	*10.83*	*0.27*	*35.59*	*341.94*	*206.17*
prss	*-2.55*	*4.32*	*0.19*	*969.52*	*1007.04*	*989.51*
mcld	*-3.52*	*3.56*	*0.14*	*0.00*	*100.00*	*22.35*
tcld	*-3.14*	*4.47*	*-0.29*	*0.00*	*100.00*	*41.59*
lcld	*-4.26*	*5.08*	*-0.48*	*0.00*	*100.00*	*28.45*
hcld	*-5.34*	*6.12*	*-0.55*	*0.00*	*100.00*	*16.36*
mslp	*-4.78*	*5.59*	*-0.94*	*1001.01*	*1040.09*	*1022.44*
pblh	*-6.92*	*5.07*	*-1.10*	*20.00*	*2422.02*	*878.05*
mofi	*-4.95*	*3.31*	*-1.22*	*0.01*	*1.41*	*0.32*
lib4	*-16.41*	*6.28*	*-1.85*	*-273.17*	*-249.14*	*-262.78*
SO_2 [μg m^{-3}]	*-12.92*	*8.56*	*-2.96*	*0.03*	*2.03*	*0.75*
lhtf	*-15.60*	*3.01*	*-5.29*	*6.52*	*100.00*	*69.11*
NO_2 [μg m^{-3}]	*-19.87*	*10.15*	*-7.21*	*1.07*	*46.87*	*7.94*
O_3 [μg m^{-3}]	*-15.99*	*2.41*	*-8.18*	*16.89*	*169.54*	*77.44*
CO [mg m^{-3}]	*-51.71*	*24.59*	*-29.07*	*0.14*	*0.36*	*0.23*

Table S12. Distribution benzene values and SHAP-based impact characteristics across subclusters within identified settings.

Cluster	*Subcluster*	*Count*	*Percentage [%]*	*benzene*	*Mean impact*	*Mean absolute impact*	*Mean normalized impact [%]*	*Mean absolute normalized impact [%]*
C0	*C0-S-1*	*403*	*12.91*	*0.28*	*-0.75*	*1.00*	*-73.50*	*98.46*
C0	*C0-S0*	*12*	*0.38*	*0.28*	*-0.75*	*0.98*	*-74.25*	*96.08*
C0	*C0-S1*	*39*	*1.25*	*0.42*	*-0.68*	*0.90*	*-66.69*	*88.14*
C0	*C0-S2*	*70*	*2.24*	*0.24*	*-0.81*	*1.05*	*-79.86*	*103.52*
C0	*C0-S3*	*41*	*1.31*	*0.30*	*-0.74*	*0.97*	*-73.26*	*95.75*
C1	*C1-S-1*	*104*	*3.33*	*0.26*	*-0.72*	*0.94*	*-71.29*	*92.56*

C1	*C1-S0*	*14*	*0.45*	*0.32*	*-0.65*	*0.94*	*-63.85*	*92.38*
C1	*C1-S1*	*16*	*0.51*	*0.23*	*-0.76*	*0.97*	*-74.87*	*95.46*
C1	*C1-S2*	*243*	*7.79*	*0.24*	*-0.76*	*0.96*	*-75.26*	*94.15*
C2	*C2-S-1*	*77*	*2.47*	*0.63*	*-0.41*	*1.00*	*-39.95*	*98.70*
C2	*C2-S0*	*57*	*1.83*	*1.33*	*0.18*	*1.24*	*17.90*	*122.11*
C2	*C2-S1*	*38*	*1.22*	*0.61*	*-0.41*	*0.99*	*-40.65*	*97.84*
C2	*C2-S2*	*34*	*1.09*	*0.38*	*-0.64*	*1.02*	*-62.67*	*100.09*
C3	*C3-S-1*	*80*	*2.56*	*0.44*	*-0.55*	*1.06*	*-53.89*	*104.03*
C3	*C3-S0*	*22*	*0.7*	*0.40*	*-0.62*	*1.23*	*-61.49*	*120.74*
C3	*C3-S1*	*18*	*0.58*	*0.39*	*-0.64*	*0.99*	*-63.20*	*97.04*
C3	*C3-S2*	*124*	*3.97*	*0.35*	*-0.68*	*1.10*	*-67.19*	*108.08*
C3	*C3-S3*	*17*	*0.54*	*0.30*	*-0.67*	*1.19*	*-65.77*	*117.00*
C3	*C3-S4*	*15*	*0.48*	*0.56*	*-0.42*	*1.10*	*-41.23*	*108.09*
C4	*C4-S-1*	*27*	*0.87*	*1.65*	*0.66*	*1.33*	*65.03*	*130.91*
C4	*C4-S0*	*208*	*6.66*	*1.81*	*0.77*	*1.40*	*75.85*	*138.15*
C4	*C4-S1*	*233*	*7.47*	*0.46*	*-0.57*	*0.96*	*-56.26*	*94.20*
C5	*C5-S-1*	*116*	*3.72*	*0.63*	*-0.37*	*0.90*	*-35.98*	*88.90*
C5	*C5-S0*	*28*	*0.9*	*0.54*	*-0.40*	*0.91*	*-39.65*	*89.72*
C5	*C5-S1*	*11*	*0.35*	*0.37*	*-0.64*	*0.98*	*-63.01*	*96.69*
C5	*C5-S2*	*11*	*0.35*	*0.70*	*-0.28*	*0.79*	*-27.41*	*77.72*
C5	*C5-S3*	*7*	*0.22*	*0.58*	*-0.44*	*0.99*	*-43.71*	*97.10*
C5	*C5-S4*	*13*	*0.42*	*0.50*	*-0.51*	*0.89*	*-49.77*	*87.14*
C5	*C5-S5*	*7*	*0.22*	*0.99*	*-0.15*	*1.02*	*-15.03*	*100.82*
C5	*C5-S6*	*52*	*1.67*	*0.59*	*-0.40*	*0.92*	*-39.43*	*90.10*
C5	*C5-S7*	*22*	*0.7*	*0.67*	*-0.31*	*0.89*	*-30.76*	*87.24*
C6	*C6-S0*	*504*	*16.15*	*3.23*	*2.19*	*3.08*	*215.69*	*303.19*
C6	*C6-S1*	*458*	*14.67*	*0.57*	*-0.43*	*0.92*	*-41.90*	*90.31*

Table S13. Predictive performance of the Extra Trees model across atmospheric settings.

	MAE	*MSE*	*RMSE*	*MAPE*	*Explained variance*	*Max Error*	R^2
Overall	*0.23*	*0.19*	*0.44*	*0.52*	*0.88*	*6.13*	*0.88*
Cluster 0	*0.14*	*0.08*	*0.27*	*0.99*	*0.61*	*3.13*	*0.61*
Cluster 1	*0.10*	*0.02*	*0.15*	*0.56*	*0.52*	*0.84*	*0.51*
Cluster 2	*0.22*	*0.14*	*0.38*	*0.48*	*0.56*	*2.98*	*0.56*
Cluster 3	*0.16*	*0.07*	*0.26*	*0.34*	*0.85*	*1.75*	*0.85*
Cluster 4	*0.19*	*0.12*	*0.34*	*0.24*	*0.86*	*3.73*	*0.86*
Cluster 5	*0.11*	*0.02*	*0.15*	*0.29*	*0.61*	*0.78*	*0.60*
Cluster 6	*0.38*	*0.45*	*0.67*	*0.24*	*0.89*	*4.75*	*0.89*
